# Ultrastructural Interactions and Genotoxicity Assay of Cerium Dioxide Nanoparticles on Mouse Oocytes

**DOI:** 10.3390/ijms141121613

**Published:** 2013-10-31

**Authors:** Blandine Courbiere, Mélanie Auffan, Raphaël Rollais, Virginie Tassistro, Aurélie Bonnefoy, Alain Botta, Jérôme Rose, Thierry Orsière, Jeanne Perrin

**Affiliations:** 1 Institut Méditerranéen de Biodiversité et d’Ecologie marine et continentale (IMBE), Biogénotoxicologie–Santé humaine et environnement (UMR CNRS 7263–FR CNRS 3098), Aix-Marseille Université, Faculté de médecine, 27 Bd Jean Moulin, Marseille 13005, France; E-Mails: r.rollais@gmail.com (R.R.); virginie.tassistro@imbe.fr (V.T.); aurelie.bonnefoy@univ-amu.fr (A.Bon.); alain.botta@imbe.fr (A.Bot.); thierry.orsiere@imbe.fr (T.O.); jeanne.perrin@imbe.fr (J.P.); 2 Department of Gynaecology, Obstetrics and Reproduction, Gynepole, AP-HM La Conception, Marseille 13005, France; 3 Centre Européen de Recherche et d’Enseignement des Géosciences de l’Environnement (CEREGE), UMR CNRS 7330, Technopôle de l’Arbois-Méditerranée BP80, 13545 Aix en Provence cedex 4, France; E-Mails: auffan@cerege.fr (M.A.); rose@cerege.fr (J.R.); 4 iCEINT, international consortium for the Environmental Implications of Nanotechnology, Technopôle de l’Environnement Arbois Méditerranée, Avenue Louis Philibert, 13545 Aix-en-Provence, France

**Keywords:** oocyte, cerium dioxide nanoparticles, genotoxicity, comet assay

## Abstract

Cerium dioxide nanoparticles (C_e_O_2_ ENPs) are on the priority list of nanomaterials requiring evaluation. We performed *in vitro* assays on mature mouse oocytes incubated with C_e_O_2_ ENPs to study (1) physicochemical biotransformation of ENPs in culture medium; (2) ultrastructural interactions with follicular cells and oocytes using Transmission Electron Microscopy (TEM); (3) genotoxicity of C_e_O_2_ ENPs on follicular cells and oocytes using a comet assay. DNA damage was quantified as Olive Tail Moment. We show that ENPs aggregated, but their crystal structure remained stable in culture medium. TEM showed endocytosis of C_e_O_2_ ENP aggregates in follicular cells. In oocytes, C_e_O_2_ ENP aggregates were only observed around the zona pellucida (ZP). The comet assay revealed significant DNA damage in follicular cells. In oocytes, the comet assay showed a dose-related increase in DNA damage and a significant increase only at the highest concentrations. DNA damage decreased significantly both in follicular cells and in oocytes when an anti-oxidant agent was added in the culture medium. We hypothesise that at low concentrations of C_e_O_2_ ENPs oocytes could be protected against indirect oxidative stress due to a double defence system composed of follicular cells and ZP.

## 1. Introduction

Engineered nanoparticles (ENPs) (size between 1 to 100 nm) are widely and extensively used in many industries such as environmental testing [1], electronics [2], textiles [3], cosmetics [4], pharmacology, and medicine (e.g., oncology, radiology) [5,6]. This growing interest is based on their large specific surface area and the novel properties specifically resulting from their small size and surface reactivity [7]. The world production of ENPs is estimated to reach 500,000 metric tons/year in 2015 [8] and raises the question of the potential long-term effects of ENP residue in the environment. Cerium dioxide nanoparticles (C_e_O_2_ ENPs) are used in the automotive industry [9], wood care applications [10], and medicine [11,12]. They are also used as additives in diesel to decrease fuel consumption and CO_2_ gas emissions [13]. According to the Health Effects Institute (HEI), C_e_O_2_ ENPs emissions are expected to reach up to 22 million pounds annually in the European Union after their introduction as diesel-additives. Consequently, the Organization for Economic Cooperation and Development (OECD) decided to include C_e_O_2_ ENPs in the priority list of the ENPs requiring evaluation. Cassee *et al.* demonstrated that environmental and human health impacts resulting from exposure to emissions with new diesel mixtures containing C_e_O_2_ ENPs were unknown and required further nanotoxicological studies [9].

Nanotoxicology is a new field in toxicology [14]. Several authors have identified insufficient nanotoxicological data [15] to perform relevant risk assessment studies. There is also a lack of standardised toxicological assays available for risk assessment of ENPs [16,17].

The effects of C_e_O_2_ ENPs on human cells remain a paradox. C_e_O_2_ ENPs have been shown to be powerful antioxidant agents [18–20], which can be used as anti-cancer treatments [21]. ENPs are also considered a promising therapy against inflammation and oxidative stress due to their free radical scavenger ability [21]. Such antioxidant properties are related to the presence of oxygen vacancies and redox transformations (C_e_^4+^/C_e_^3+^) occurring at the surface of C_e_O_2_ ENPs. Conversely, several publications have demonstrated harmful effects of C_e_O_2_ ENPs on somatic cells [22], aquatic organisms [23–26], or bacteria [27]. C_e_O_2_ ENPs are able to penetrate through cell membranes or can be internalised by endocytosis [22] to induce oxidative stress, inflammation, cytotoxicity and genotoxicity [28,29]. In a previous work, Auffan *et al.* demonstrated the internalisation of C_e_O_2_ ENPs using human dermal fibroblasts and DNA damage related to oxidative stress [30]. Such stress was related to the biotransformation and surface reactivity of the C_e_O_2_ ENPs in the biological medium. These paradoxical results demonstrate that the evaluation of ENP safety is a public health priority requiring basic research concerning interactions of ENPs with ecosystems [31] and human health [16,32].

Exogenous compounds are able to accumulate in ovaries. For example, mercury can accumulate after application of skin-lightening creams [33]. However, the biodistribution of ENPs in ovaries is unknown [34,35]. Several publications have focused on the effects of ENPs in the reproduction of aquatic organisms [36–41]. Few studies focused on mammalian male germ cells and spermatogonia [42,43], and a limited number of studies have addressed the effects of ENPs on human germ cells [44]. Chaudhury *et al.* suggested that ovarian exposure to C_e_O_2_ ENPs by intraperitoneal injection had no adverse effect on the rate of mature oocytes. However, this study provided no information regarding genotoxicity of C_e_O_2_ ENPs on oocytes [45]. The current work is the first study focusing on the mechanisms of interactions and biological effects between mouse oocytes and C_e_O_2_ ENPs. The novelty of our approach is a combination of *in vitro* genotoxicity testing with a thorough physico-chemical characterisation of C_e_O_2_ ENPs in the biological media of oocytes.

## 2. Results and Discussion

### 2.1. Physico-Chemical Behaviour of the C_e_O_2_ ENPs in the Oocyte Culture Medium M16

**Figure 1:**
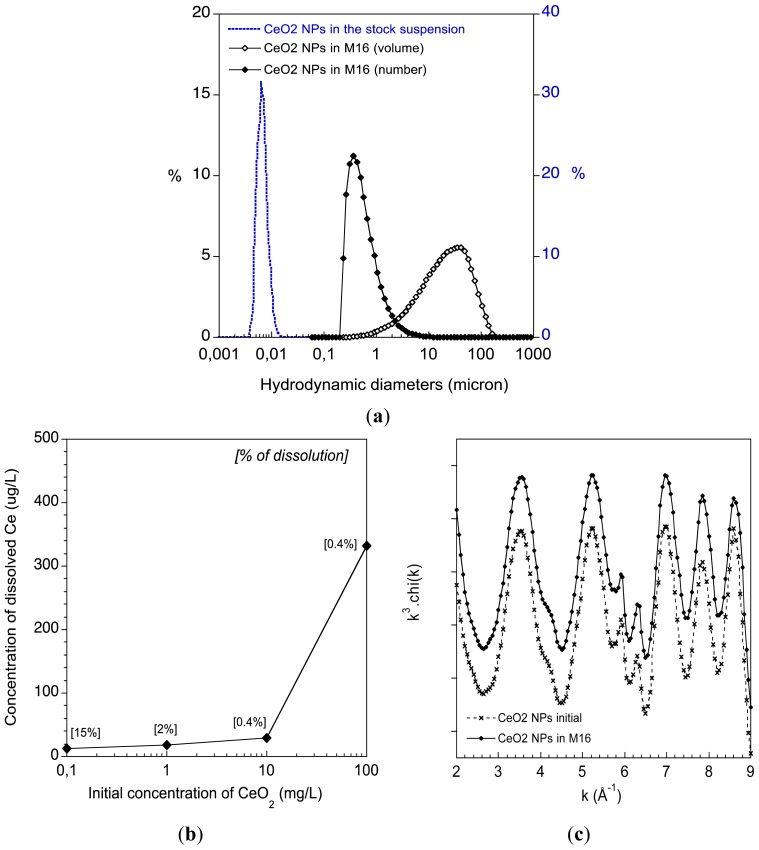
(**a**) Aggregation stage of the C_e_O_2_ ENPs in the M16 medium. Distribution of the hydrodynamic diameters of the C_e_O_2_ ENPs in their stock suspension and after 2 h in the M16 medium (expressed as a volume or number distribution); (**b**) Chemical stability of the C_e_O_2_ ENPs in M16. Dissolution of C_e_O_2_ ENPs after 2 h in the abiotic M16 determined by ICP-MS; (**c**) Structural stability of the C_e_O_2_ ENPs in M16. EXAFS at the C_e_ L3-edge of the C_e_O_2_ ENPs before and after 2 h of incubation within the abiotic M16 medium.

The colloidal stability of the C_e_O_2_ ENPs was studied by dynamic light scattering (DLS) after 2 h of incubation in the abiotic M16 culture medium. While the ENPs are stable in their stock suspension, a significant aggregation occurs in M16 (Figure 1a). Aggregates with hydrodynamic diameters (volume distribution) centred at ~35 μm quickly formed. When expressed as a number distribution most of these aggregates have hydrodynamic diameters of approximately 350 nm. It is noteworthy that this number distribution is based on several assumptions (e.g., shape, density of the aggregates) but it highlights that most of the C_e_O_2_ ENPs interacted with the oocytes as small aggregates. The dissolution of the C_e_O_2_ ENPs in the M16 was studied by ICP-MS (Figure 1b). After 2 h, less than 30 μg/L of dissolved C_e_ was measured in the abiotic M16 media for an initial C_e_O_2_ concentration below 10 mg/L. For 100 mg/L of initial C_e_O_2_ ENP concentration, 331 μg/L of dissolved C_e_ was measured in solution (0.4% of the total C_e_ content). The release of C_e_ ions was low, typically <0.4% of the initial concentrations. EXAFS (Extended X-ray Absorption Fine Structure) was used to study the crystal structure of the C_e_O_2_ ENPs (*i.e*., the number, nature and distances of atoms surrounding Ce from 0 to 5 Å) after incubation in abiotic M16 (Figure 1c). The experimental spectra of C_e_O_2_ ENPs before and after 2 h of incubation in M16 perfectly superimpose, indicating that the atomic structure of the C_e_O_2_ ENPs is not affected (Figure 1c). Such local-scale stability suggests that the ENPs surface interaction with macromolecules from the M16 (proteins) is not associated with major surface complexation or reduction of C_e_^4+^ into C_e_^3+^. Notably, EXAFS is not sensitive to minor Ce species (*i.e*., <10%). Therefore, the detection of less than 0.4% C_e_ dissolution is not contradictory with the EXAFS main result concerning C_e_O_2_ structure.

After 2 h of incubation with the abiotic M16 medium the C_e_O_2_ ENPs can be considered structurally stable and have a slow release of dissolved C_e_. However, the ENPs have a strong colloidal destabilisation, and most of the aggregates are approximately 350 nm.

### 2.2. Transmission Electron Microscopy (TEM) Study of C_e_O_2_ ENPs Internalisation

**Figure 2:**
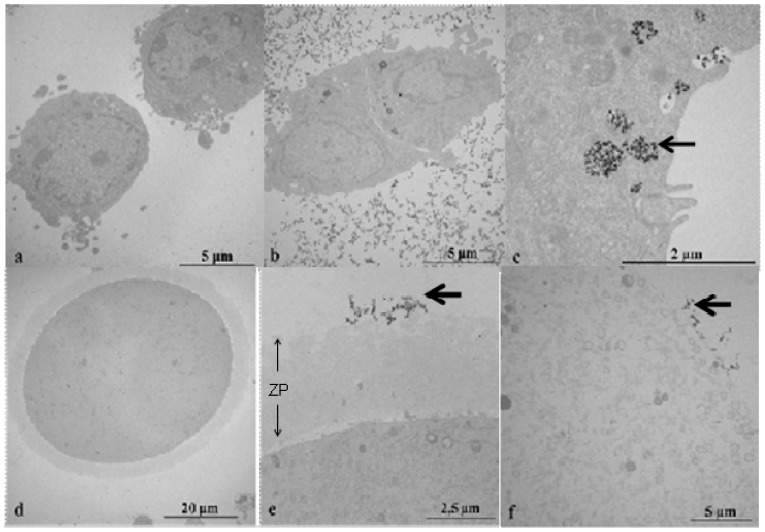
Transmission electron microscopy image of follicular cells and oocytes exposed to C_e_O_2_ ENPs. (**a**) Unexposed follicular cells; (**b**) Follicular cells exposed to C_e_O_2_ NPs (wide shot); (**c**) Follicular cells exposed to C_e_O_2_ ENPs (close-up); (**d**) Unexposed oocyte surrounded by zona pellucida; (**e**) Oocyte surrounded by zona pellucida (ZP+) exposed to C_e_O_2_ ENPs; (**f**) Oocyte not surrounded by zona pellucida (ZP−) exposed to C_e_O_2_ ENPs.

The TEM study showed the internalisation of C_e_O_2_ ENP aggregates by endocytosis in follicular cells (Figure 2b,c). In oocytes surrounded by zona pellucida (ZP+) incubated with C_e_O_2_ ENPs, the C_e_O_2_ ENP aggregates were only observed around the zona pellucida (ZP) (Figure 2e). In oocytes not surrounded by zona pellucida (ZP−), we did not observe any C_e_O_2_ ENPs in the oocyte cytoplasm (Figure 2f).

### 2.3. C_e_O_2_ ENPs Induced DNA Damage in Follicular Cells

**Figure 3:**
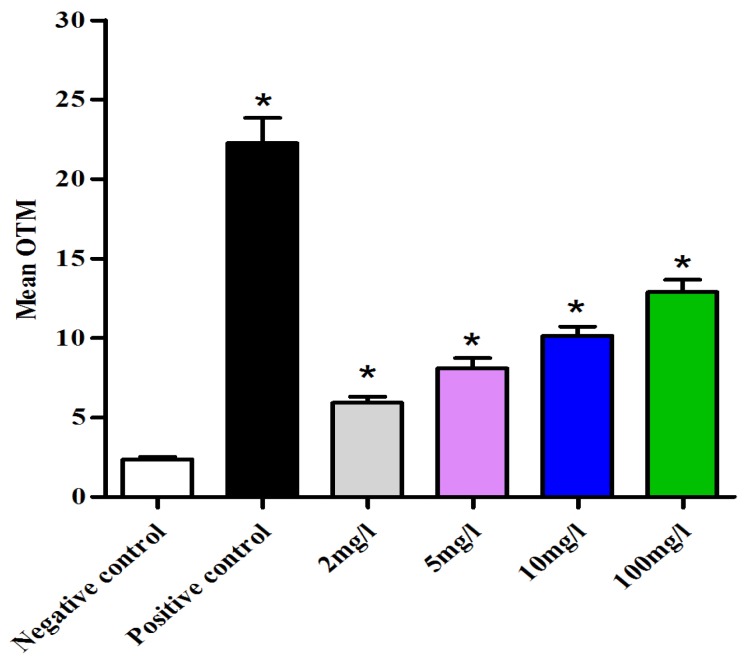
Genotoxicity assessment of C_e_O_2_ ENPs on follicular cells by comet assay. *p ≤ 0.05.

Although several genetic toxicology tests have been validated for chemicals according to the Organisation for Economic Co-operation and Development (OECD) test guidelines, the relevance of these assays for nanoparticulate materials remains to be determined. To address this issue, the OECD has established current projects designed to evaluate the relevance and reproducibility of safety hazard tests for representative nanomaterials, including genotoxicity assays [17]. No reference gene mutation test has been described for oocytes. The genotoxicity of C_e_O_2_ ENPs on mouse oocytes and on follicular cells was assessed with the protocol previously described in our research laboratory [46]. A comet assay was used in this study because it is very sensitive and allows the detection of DNA double and single-strand breaks in individual eukaryotic cells. The principle underlying the comet assay is that denatured DNA fragments can be measured migrating out of the cell nucleus during electrophoresis [47]. The image obtained is a “comet” with a distinct head consisting of intact DNA and a tail containing relaxed DNA loops or broken pieces of DNA [48]. The comet assay quantifies DNA damage with the Olive Tail Moment (OTM = % DNA in the tail × length of the comet tail). At least 350 follicular cells were studied for each condition using the comet assay. Significant dose-dependent DNA damage was observed in follicular cells exposed to 2, 5, 10 and 100 mg/L of C_e_O_2_ ENPs with OTM, respectively at 5.9 ± 0.4; 8.1 ± 0.6; 10.1 ± 0.6 and 12.9 ± 0.7 *vs.* 2.3 ± 1.6 for the negative control group and 22.2 ± 1.6 for the positive control group (Figure 3).

### 2.4. DNA Damage Assay in Oocytes

**Figure 4:**
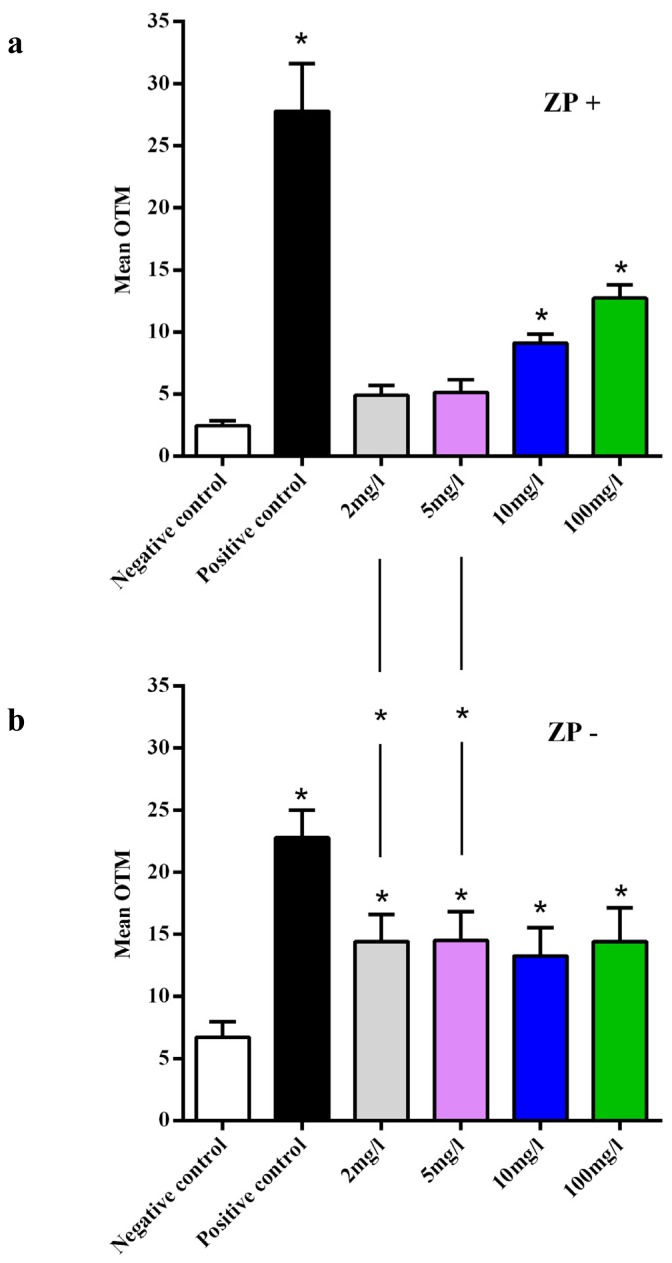
Genotoxicity assessment of C_e_O_2_ ENPs on mouse oocytes by comet assay (**a**) Oocytes surrounded by zona pellucida (ZP+); (**b**) Oocytes not surrounded by zona pellucida (ZP−). *p ≤ 0.05.

In ZP+ oocytes, we did not observe significant DNA damage at 2 mg/L (OTM = 4.8 ± 0.8) and at 5 mg/L (OTM = 5.1 ± 1) compared to 2.4 ± 0.4 for the negative control group and 27.8 ± 3.8 for the positive control group (Figure 4a). The two highest exposure concentrations induced a significant increase of OTM values: 9.1 ± 0.74 at 10 mg/L and OTM = 12.7 ± 1.1 at 100 mg/L. In ZP− oocytes, we observed a significant increase of DNA damage after C_e_O_2_ ENPs exposure, with OTM, respectively, at 14.4 ± 2.1, 14.5 ± 2.3, 13.3 ± 2.2 and 14.4 ± 2.7 for 2, 5, 10 and 100 mg/L *vs.* 6.7 ± 1.3 for the negative control group and 22.8 ± 2.2 for the positive control group (Figure 4b).

### 2.5. C_e_O_2_ ENPs and Anti-Oxidant

**Figure 5:**
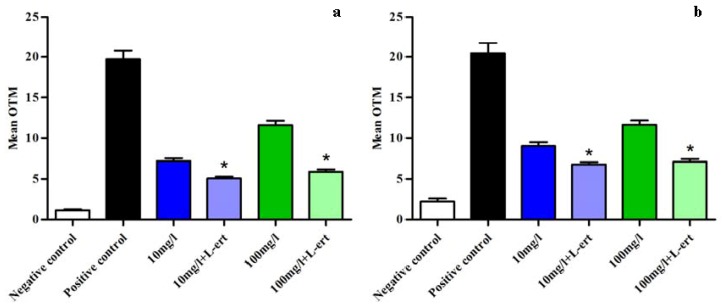
Reduction of DNA damage induced by C_e_O_2_ ENPs using the comet assay after adding an anti-oxidant agent (l-ert = l-ergothioneine) in culture media. (**a**) Follicular cells; (**b**) Mouse ZP+ oocytes. *p ≤ 0.05.

When l-ergothioneine was added in culture medium before incubation of ZP+ oocytes and follicular cells with C_e_O_2_ ENPs, DNA damage decreased significantly in both cell types. At 10 mg/L and 100 mg/L, OTM in the groups treated with l-ergothioneine were 5.1 ± 0.2 *vs.* 7.2 ± 0.3, respectively, in follicular cells with l-ergothioneine and 5.9 ± 0.3 *vs.* 11.6 ± 0.5 without l-ergothioneine (Figure 5a). In ZP+ oocytes, OTM were respectively 2.8 ± 0.5 *vs.* 6.7 ± 0.3 with l-ergothioneine (*p* < 0.05) and 7.1 ± 0.3 *vs.* 11.6 ± 0.5 without l-ergothioneine (*p* < 0.05) (Figure 5b). The OTM in negative and positive control groups were respectively 1.1 ± 0.1 and 19.7 ± 1.1 in follicular cells and 2.2 ± 0.3 and 20.4 ± 1.3 in ZP+ oocytes.

### 2.6. Discussion

To our knowledge, this is the first study focused on the genotoxicity of C_e_O_2_ ENPs in oocytes. The genotoxicity of C_e_O_2_ ENPs depends on the physico-chemical properties of the cell’s environment because it determines aggregation, redox modifications, and surface adsorption [49]. To safely use ENPs future research on the properties of ENPs must focus on biochemical and physical interactions between ENPs and the environment. In the conclusions of a working group from the International Life Sciences Institute Research Foundation/Risk Science Institute Nanomaterial Toxicity Screening Working Group, Oberdorster *et al.* suggested a toxicity assay of ENPs evaluating their potential effects on human health should involve a multidisciplinary approach including physico-chemical characterisation as well as *in vitro* and *in vivo* assays [16]. For the *in vitro* genotoxicity assay, we used the comet assay, which is a widely used test in toxicology and well-adapted to mouse oocytes [46]. Given the new and unique physico-chemical properties of ENPs, standardised genotoxicity assays must be adapted to respond to risk assessment questions. The dose-response relationship is a function of the physico-chemical behaviour and surface reactivity of different classes of ENPs [16].

#### 2.6.1. Are Oocytes Protected from C_e_O_2_ ENP Induced Oxidative Stress and DNA Damage by Follicular Cell Endocytosis and Zona Pellucida Trapping?

Our study demonstrated that the intracellular delivery of C_e_O_2_ ENPs into follicular cells was possible by endosomal trapping. Endosomal trapping is a mechanism developed by cells to protect themselves from foreign organisms [50]. A combination of DLS and TEM analyses showed that despite aggregation in the exposure media, C_e_O_2_ ENPs were internalised by follicular cells. The kinetics of intracellular uptake most likely depends on the size of ENP aggregates, as shown by Rejman *et al.* [51]. While the internalisation of ENPs can be useful in biomedical applications [52], we hypothesised it could induce the oxidative stress observed in follicular cells in our experimental conditions. This oxidative stress was indirectly demonstrated by the significant decrease of DNA damage in follicular cells after the addition of l-ergothioneine in the exposure medium.

Regarding the oocytes, TEM images did not show any intracellular delivery into oocytes. However, C_e_O_2_ ENPs were observed trapped on the ZP surface outside the oocyte membrane. The ZP could act as a mechanical barrier excluding C_e_O_2_ ENPs, which decreases their direct harmful effects or decreases the indirect effects of C_e_^3+^ ions released from the ENPs in the M16 culture medium. Following oocyte exposure to 2 and 5 mg/L, we did not observe significant DNA damage in ZP+ oocytes. Conversely, DNA damage was significantly increased in ZP− oocytes. At high ENP concentrations, DNA damage was observed in oocytes both with and without zona pellucida. This result suggests that the defence system preventing DNA damage and oxidative stress was overwhelmed. This hypothesis requires further evaluation by *in vivo* studies.

The DNA damage was dose-dependent in follicular cells and in oocytes surrounded by zona pellucida. However, no dose-response relationship was observed in oocytes lacking ZP. The OTM values obtained at the lowest C_e_O_2_ ENP concentrations tested in ZP− oocytes were similar to the highest dose-related effect of C_e_O_2_ ENPs tested in ZP+ oocytes. Thus, the dose-related effect of C_e_O_2_ ENPs observed with follicular cells and ZP+ oocytes was not present in ZP− oocytes. This result may be because DNA damage quantified by the comet assay was maximal at the lowest tested concentration in ZP− oocytes.

#### 2.6.2. Mechanisms of Oxidative Stress Induced by C_e_O_2_ ENPs

In ZP+ oocytes, exposure to C_e_O_2_ ENPs statistically increased DNA damage only at high concentrations (10 and 100 mg/L), while in ZP− oocytes DNA damage was observed at all the tested concentrations. We observed that the DNA damage significantly decreased in ZP+ oocytes after addition of an anti-oxidant agent. Consequently, we hypothesised that C_e_O_2_ ENPs induced oxidative stress in oocytes and that ZP may protects oocytes against oxidative stress at low concentrations of C_e_O_2_ ENPs.

Several biophysico-chemical reactions occurring at the ENP/biological interface are able to influence the surface properties of ENPs and consequently their biological effects [53]. Changes of the redox state of the ENPs, their dissolution, the adsorption of organic matter, and the aggregation state (size and density) can influence the exposure and toxicological impacts of ENPs. Dynamic interactions between ENPs and cells, membranes, DNA, and intracellular organelles can lead to favourable or adverse biological effects according to ENP properties and biotransformation [54]. For instance, Asati *et al.* showed that the internalisation profile in normal and cancer cell lines and the cytotoxicity potential depended on the C_e_O_2_ ENPs’ surface charge [22]. Zeyons *et al.*, (2009) have shown that C_e_O_2_ ENPs induced cytotoxicity on Escherichia coli via a direct mechanism of bio-reduction requiring a close contact between ENPs and the cell membranes [55]. Additionally, the authors found that C_e_O_2_ ENPs induced indirect cytotoxicity on Synechocystis by extracellular polymeric substances preventing direct cellular contacts with the ENPs in addition to the acidity of the ENP stabilising agent.

Indirect biological effects were observed in our experimental conditions. Our results suggest that when mature oocytes are exposed to low concentrations of C_e_O_2_ ENPs, follicular cell endocytosis and zona pellucida trapping protected oocytes by counteracting oxidative stress that prevents DNA damage in oocytes. However, we observed DNA damage at high concentrations of C_e_O_2_ ENPs. The DNA damage could be caused by a direct effect of the C_e_O_2_ ENPs on oocytes or by indirect effects of C_e_^3+^ ions released from the ENPs in the M16 culture medium. Based on our results, it is likely that the follicular cells and zona pellucida may prevent direct contact between the C_e_O_2_ ENPs and the oocytes. The C_e_^3+^ ions could diffuse through the zona pellucida to indirectly stress the cells. These findings demonstrate the importance of studying the physico-chemical behaviour and biotransformation of ENPs to thoroughly understand the mechanisms of genotoxicity towards germinal cell lines.

## 3. Experimental Section

### 3.1. Chemical Agents

All chemicals were from Sigma (St. Quentin–Fallavier, France) unless stated otherwise.

### 3.2. C_e_O_2_ ENPs Physico-Chemical Characterisation in Culture Medium

The C_e_O_2_ ENPs (Rhodia^®^, Courbevoie, France) used in this study are pseudo-spherical crystallites of cerianite with an average size of 3 nm (total number of clusters measured: 70). These ENPs are uncoated and are dispersed in pure water with an average hydrodynamic diameter of ~8 nm (Figure 1a). The size, shape and mineralogy were characterised by Transmission Electron Microscopy (TEM) (using a JEOL 2010F at 200 kV). Dynamic light scattering (DLS) (using a nano ZS and a Mastersizer S, Malvern Instruments SA, Orsay, France) was used to determine their aggregation states before and after 2 h of incubation in the M16 media. The dissolution of the C_e_O_2_ ENPs in the M16 medium was assessed using ICP-MS. Briefly, the C_e_O_2_ ENPs were incubated for 2 h in the abiotic M16. After incubation, the suspension was ultra-centrifuged (200.000 g for 1 h) and the supernatant was analysed by ICP-MS. The solid phase of the centrifugation was freeze-dried and analysed by X-ray absorption spectroscopy (XAS) for structural characterisation. XAS at the Ce L3-edge (5723 eV) was performed on the XAFS 11.1 beamline [56] at the ELETTRA synchrotron (Trieste, Italy). Samples were diluted in boron nitride, pressed to thin pellets, and analysed in transmission mode. The spectra were compiled from the merge of three scans, and the energy was calibrated using a C_e_O_2_ standard reference. EXAFS (Extended X-ray Absorption Fine Structure) data were obtained after performing standard procedures for pre-edge subtraction, normalisation, polynomial removal, and wave vector conversion using the IFEFFIT software package [57].

### 3.3. Animals

Prepubescent 4-week old female mice CD1 (Charles River Laboratory, L’Arbresle, France) were housed in a temperature and light controlled room with free access to food and water. Institutional Review Board approval n° 12-18042012 was obtained after submission of the experimental protocol and animal handling procedures to the National Ethics Committee on Animal Experimentation.

### 3.4. Oocytes Isolation

Mice were injected intraperitoneally with 0.1 mL of 10 IU Pregnant Mare Serum Gonadotropin. Three days later, they received an additional injection with 0.1 mL of 5 IU of Human Chorionic Gonadotropin. Sixteen hours later, mice were sacrificed by cervical dislocation [58] and oviducts were collected. Intact cumulus masses were released from excised oviducts, and decoronisation was performed after incubation with hyaluronidase (10 mg/mL). Digestion of zona pellucida needed for TEM evaluation and genotoxicity assays was performed with acidic Tyrode’s solution. The oocytes were then washed three times in M2 medium. Approximately 25–30 mature oocytes were released for each mouse. Each experimental condition included at least 40 mature oocytes.

### 3.5. Transmission Electron Microscopy (TEM)

Follicular cells and oocytes with or without the zona pellucida (ZP) were incubated for 2 h *in vitro* with C_e_O_2_ ENPs (100 mg/L) and the potential internalisation was studied by TEM (transmission electronic microscopy). After incubation, follicular cells and oocytes were washed twice in cacodylate buffer (0.1 M, pH = 7.4) fixed in 2.5% glutaraldehyde and post-fixed in osmium tetroxide 2% in the same buffer. After centrifugation, the pellets were dehydrated in graded alcohol solutions and embedded in Embed-812 kit using a standard procedure. Ultrathin sections (60–70 nm) were counterstained with uranyl acetate and lead citrate before observation with a JEOL/JEM 1400 electron microscope at 80 kV.

### 3.6. Exposure Conditions for Genotoxicity Assay

We used the comet assay on mature mouse oocytes as described in a previous work [46]. Oocytes (*n* = 40 for each group) and follicular cells (at least 100 for each group) were exposed to 4 concentrations of C_e_O_2_ ENPs (2, 5, 10 and 100 mg/L) in the M16 medium at 37 °C and 5% CO_2_ during 2 h. Two groups of oocytes were studied with (ZP+) or without the zona pellucida (ZP−). Negative control oocytes and follicular cell groups were incubated for 2 h in the M16 medium. For the positive control group, oocytes were incubated at 37 °C with 5% CO_2_ in M16 medium for 2 h. The cells were placed at the end of incubation in a 250 μM hydrogen peroxide (H_2_O_2_) solution for 5 min at 4 °C in the dark. Each condition was replicated three times.

### 3.7. Incubation of Oocytes with an Anti-Oxidant

For the conditions that induced DNA damage for the comet assay each experiment was repeated with an anti-oxidant added in the M16 medium. For the conditions with 10 mg/L and 100 mg/L of C_e_O_2_ ENPs, l-ergothioneine (5 mM) was added to the culture medium with follicular cells and ZP+ oocytes. l-ergothioneine has a well-known anti-oxidant activity that can scavenge hydroxyl radicals and inhibit the generation of hydroxyl radicals from hydrogen peroxide [59]. These two incubation conditions were compared with 2 groups (10 mg/L and 100 mg/L of C_e_O_2_ ENPs) without l-ergothioneine and to the same negative and positive control groups previously described. Each condition was repeated three times with at least 100 follicular cells and 40 matures oocytes were analysed by the comet assay.

### 3.8. Main Outcome Measures and Statistical Analysis of the Comet Assay

For each tested condition, all the oocyte and follicular cell images were analysed by the validated Komet software (version 6.0; Andor. Bioimaging, Nottingham, UK). DNA damage was expressed as Olive Tail Moment (OTM, arbitrary units), which is the association of tail length and DNA% contained in tail [48]. The results were expressed as the mean values ± SEM and analysed by ANOVA followed by Fisher LSD post-hoc test using Statview^®^ 5.1 for Windows (Abacus Concepts, Berkeley, CA, USA). The statistical significance was set at *p* < 0.05.

## 4. Conclusions

Our study was a first examining the mechanisms of interactions between C_e_O_2_ ENPs and germ cells. Our results cannot be extrapolated to *in vivo* function of C_e_O_2_ ENPs by inhalation, but offer a tool to understand the mechanisms of possible interactions between C_e_O_2_ ENPs and female germ cells. Our results suggested that when mature oocytes are exposed to low concentrations of C_e_O_2_ ENPs, follicular cell endocytosis and zona pellucida trapping could protect oocytes by decreasing oxidative stress and DNA damage. At low concentrations (more expected in environmental exposure), these preliminary data seem to us reassuring as for the possible impact of an environmental exposure of C_e_O_2_ ENPs on mature oocytes. However, at high concentrations this defence system may be insufficient to prevent induction of oxidative stress leading to DNA damage. Moreover, oocytes exposed at earlier stages of maturation, with no or immature ZP and fewer follicular cells, could be more vulnerable to DNA damage induced by C_e_O_2_ ENPs. Given the impact demonstrated by the C_e_O_2_ ENPs on the reproduction of the aquatic organisms [23,24] and the hypothesis of a possible bioaccumulation in ovary [33], we plan to study interactions between C_e_O_2_ ENPs and oocytes. *In vitro* studies will help us to determine the mechanisms of ENP interactions under controlled conditions that represent *in vivo* situations.
